# Impella-Assisted Intracoronary Lithotripsy of Severely Calcified Left Main Coronary Artery Bifurcation for NSTEMI With Cardiogenic Shock

**DOI:** 10.7759/cureus.14772

**Published:** 2021-04-30

**Authors:** Yashwant Agrawal, Dominika Zoltowska, Jean-Yves R Nazroo, Abdul R Halabi

**Affiliations:** 1 Interventional Cardiology, St. Joseph Mercy Oakland Hospital, Pontiac, USA; 2 Interventional Cardiology, Ascension Health, Kalamazoo, USA; 3 Cardiology, University of Florida College of Medicine, Jacksonville, USA; 4 Internal Medicine, St. Joseph Mercy Oakland Hospital, Pontiac, USA

**Keywords:** case report, shockwave intravascular lithotripsy, impella, nstemi, acs, cardiogenic shock, cad, pci

## Abstract

High calcification of coronary artery plaque is a frequent cause of suboptimal stent expansion, which can result in stent thrombosis and restenosis. Shockwave intravascular lithotripsy (S-IVL) represents a new frontier in the treatment of highly calcified coronary lesions. It can be an excellent alternative to intracoronary atherectomy in extremely high-risk lesions. We present a case of a 57-year-old man with known severe coronary artery disease (CAD) who presented with non-ST segment elevation myocardial infarction (NSTEMI), cardiogenic shock and was successfully treated with impella-assisted shockwave-intravascular lithotripsy permitting successful percutaneous intervention of a high-risk left main coronary artery (LMCA) bifurcation in-stent restenosis.

## Introduction

For heavily calcified coronary lesions, traditional percutaneous coronary interventions (PCIs) include compliant balloons, non-compliant (NC) balloons, specialty balloons (cutting balloon, scoring balloon), and atherectomy (laser, rotational, and orbital). Intimal calcification remains the largest limiting factor for stent expansion, with ensuing increased risks of stent thrombosis and in-stent restenosis. Shock wave lithotripsy (SWL) technology is established for nephrolithiasis and peripheral arterial disease (PAD) with consistent safety. This therapy has been safely adapted for shockwave intravascular lithotripsy (S-IVL) for coronary interventions in recent clinical trials.

## Case presentation

**Figure 1:**
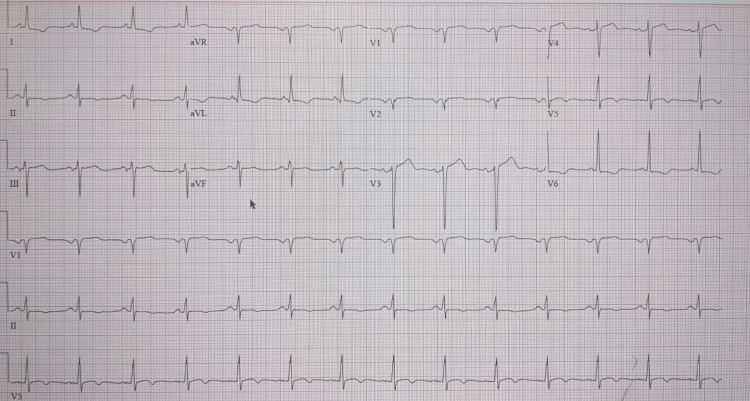
Electrocardiogram with anteroseptal Q-waves and diffuse ST-T changes.

A 57-year-old Caucasian man with a history of coronary artery disease (CAD) with multi-vessel PCI to the ostial and distal left main coronary artery (LMCA) bifurcation three months ago, chronic obstructive pulmonary disease (COPD), hyperlipidemia, severe PAD with right above the knee amputation, and stage IIIa lung cancer status - post-pneumonectomy and chemotherapy was referred to our institution from a nearby hospital for coronary revascularization. Initially, he presented to their facility with loss of consciousness and respiratory distress. Initial vitals were significant for BP 76/42 mm Hg, HR of 96 bpm, and significant hypoxia requiring mechanical ventilation. Electrocardiogram (EKG) revealed normal sinus rhythm with anterior septal q-waves and diffuse ST-T abnormalities (Figure 1). Their investigations revealed non-ST segment elevation myocardial infarction (NSTEMI) with a troponin of 12.4 ng/ml. He was initiated on norepinephrine 20 mcg/min and dobutamine 7.5 mcg/kg/min to maintain a mean arterial pressure of 65 mm Hg. Coronary angiography revealed a calcified 95% stenosis of the distal LMCA that extended into the left anterior descending (LAD) and left circumflex (LCx) coronary arteries. The right coronary artery (RCA) showed 50% non-obstructive disease (images of the angiogram from the outlying facility were not provided to us). Transthoracic echocardiogram (TTE) revealed a left ventricular ejection fraction (LVEF) of 38%, with hypokinesis of the apex, distal anterior, and anterolateral walls. An intra-aortic balloon pump (IABP; Maquet, Fairfield, NJ) was inserted and the patient was transferred to our institution for coronary revascularization. Three months prior to presenting to the outside facility, he was hospitalized for chest pain and shortness of breath. He underwent PCI with the placement of one drug-eluting stent (DES) in the ostial LMCA and two DES in the distal LMCA with coverage of the ostial and proximal LAD (details of the intervention from the outlying facility were not provided to us).

Upon arrival to our institution with an IABP (left femoral access) in place, he was also on mechanical ventilation. Vitals were significant for a blood pressure of 84/60 mmHg while on norepinephrine 30 mcg/min and dobutamine 7.5 mcg/kg/min with a heart rate of 90/min. Pertinent physical examination findings included bibasilar crackles, feeble bilateral femoral pulses, and a right above-the-knee amputation. The patient was sedated, yet his cognitive and neurological conditions were deemed to be intact as evaluated by the medical ICU team. A heart team approach was undertaken, and the cardiothoracic surgical team deemed that he was not a candidate for coronary artery bypass graft surgery, given his significant comorbidities, precarious hemodynamic status, and multiple prohibitive risk factors. Laboratory results revealed potassium of 3.3 mEq/L, WBC of 13.2/mm3, Hb 9.7 of g/dl, troponin of 1.34 ng/ml, and BNP of 900 pg/ml. Chest X-ray demonstrated prominent right lung interstitial markings. EKG remained unchanged from the initial EKG.

The decision was made to proceed with LV Impella-assisted high-risk PCI (Abiomed, Danvers, MA). The indications, benefits, risks, and rationale were discussed with the patient's family, and they agreed to proceed with this high-risk procedure. The patient was then taken to the cardiac catheterization lab. Two perclose sutures (Abbott Cardiovascular, Santa Clara, CA) were deployed for left femoral pre-closure in preparation for the insertion of the Impella CP 4.0 device. The IABP was then exchanged for the Impella, which was successfully placed in the LV via left femoral arterial access and set to P7.

The right radial artery was accessed, and a 7-French EBU 3.5 guide catheter was used to engage the LMCA. A coronary angiogram revealed a 95% stenosis of the distal LMCA, extending into the LAD and LCx (Videos 1 and 2). Two separate interventional wires were introduced in the LAD and the LCx. Kissing percutaneous transluminal coronary angioplasty (PTCA) of the distal LMCA bifurcation towards the LAD and LCx was performed using 3.0 × 12 mm^2^ and 2.5 × 12 mm^2^ NC balloons, respectively. A 4.0 × 15 mm^2^ NC balloon was used to perform balloon angioplasty of the LAD lesion. High-risk coronary atherectomy was avoided given the history of recent PCI with stenting three months ago. 

**Video 1:** Coronary angiogram demonstrating 95% stenosis of the distal LMCA and LCx in right anterior oblique view. LMCA: left main coronary artery, LCx: left circumflex.

**Video 2:** Coronary angiogram revealing 95% stenosis of the distal left main extending into the ostial LAD and ostial LCx in the left anterior oblique view. LAD: left anterior descending, LCx: left circumflex.

Current results with PTCA were suboptimal (Video 3), as there was persistent stent recoil and suspected stent under-expansion. S-IVL was therefore considered as an off-label option to optimize stent expansion and luminal gain. Informed consent had been obtained for S-IVL from the patients’ family. A 3.5 × 40 mm^2^ shockwave balloon was advanced into the distal LMCA and the LAD and a total of 80 pulses were administered with the balloon inflated to between 3 and 6 atmospheres (Video 4) with lesion modification with yielding of the extraluminal calcification. Following S-IVL angiogram was performed (Video 5), and then we proceeded with PTCA of the distal LMCA to ostial LCx which was also performed with a 3.0 × 15 mm^2^ angiosculpt balloon (Koninklijke Phillips, Nevada).

**Video 3:** Coronary angiogram post PTCA with suboptimal results. PTCA: percutaneous transluminal coronary angioplasty.

**Video 4:** Shockwave intravascular lithotripsy. A 3.5 mm × 40 mm shockwave balloon in the distal LMCA to LAD is demonstrated. LMCA: left main coronary artery, LAD: left anterior descending.

**Video 5:** Coronary angiogram performed post-S-IVL resulted in lesion modification. S-IVL: shockwave intravascular lithotripsy.

**Figure 2:**
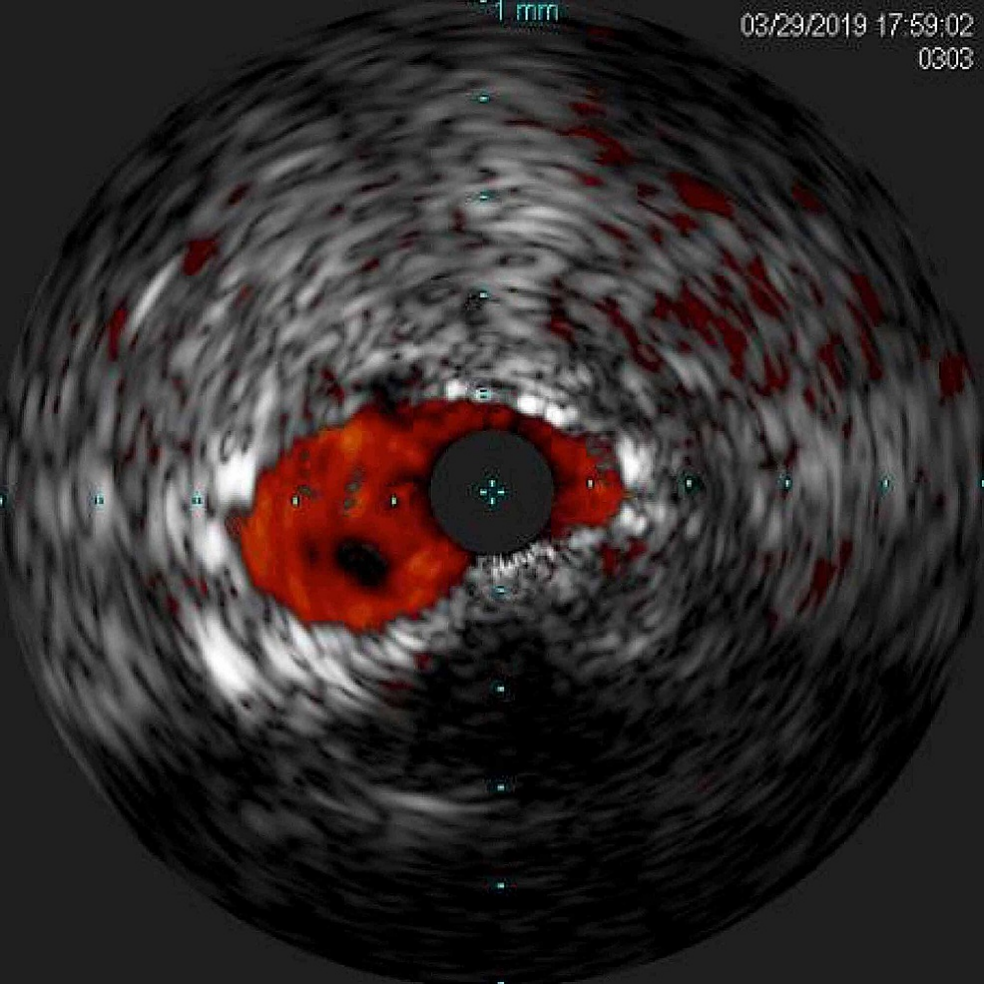
Intravascular ultrasound demonstrating an under-expanded stent in the distal LMCA. LMCA: left main coronary artery.

Intravascular ultrasound (IVUS) initially revealed an under-expanded stent in the distal LMCA (Figure 2). An IVUS guided 4.0 × 23 mm^2^ DES was deployed in the LMCA into the LAD, overlapping with the prior stents. Subsequently, it was post-dilated initially with a 4.5 × 20 mm^2^ NC balloon followed by a 5.0 × 20 mm^2^ NC balloon. Finally, there was TIMI-III flow in the LAD as well as LCx with less than 40% residual stenosis in the ostial LAD and the ostial LCx (Videos 6 and 7). The impella CP device was then removed, and both perclose sutures were deployed and tightened, with excellent vascular hemostasis. The patient tolerated the procedure well without complications or major adverse cardiovascular events (MACE). Activated clotting time levels were maintained therapeutically with the utilization of intravenous heparin. He was extubated on the following day. His Hb level was stable and his Cr level returned to baseline at 1.4 mg/dL. He was weaned off pressors successfully and he was started on guideline-directed medical therapy with aspirin and prasugrel (10 mg/day) being the dual antiplatelet agents. He remained stable from a neurological standpoint. He was discharged to an outpatient rehabilitation facility after a total of five days of hospitalization. At a two-week follow-up, the patient was doing well and had returned to baseline independence. A TTE performed at three-month follow-up demonstrated an improved LVEF of 55%. He continues to do well on follow-up in our outpatient clinic. He was advised to remain on long-term dual antiplatelet therapy (DAPT) and high-dose high-intensity statin therapy.

**Video 6:** A 4.0 mm × 23 mm DES post-dilated initially with a 4.5 mm × 20 mm NC balloon followed by a 5.0 mm × 20 mm NC balloon. DES: drug-eluting stent, NC: non-compliant.

**Video 7:** TIMI-III flow in the LAD as well as LCx with less than 40% residual stenosis in the ostial LAD and less than 40% stenosis of the ostial LCx. LAD: left anterior descending, LCx: left circumflex.

## Discussion

SWL is an established therapy that is actively used for the management of nephrolithiasis. Recently, the therapeutic principle has been used intravascularly for intimal calcification resulting in limitation of coronary stent expansion in coronary revascularization, as in our patient [1-4]. As described in the literature, the sonic pressure waves are tissue-selective and evenly distributed, disrupting both superficial and deep calcium within vascular plaques via calcium fracture “while minimizing injury and maintaining the fibroelastic components of the vessel wall" [5]. Therefore, this facilitates the optimal deployment of the stent where extensive calcification would have otherwise precluded successful stent apposition and expansion, the latter being the most important predictor of stent thrombosis and restenosis following PCI [1-5].

Such lesions have also been understood to damage DES material, which also contributes to the failure of PCI [5]. In the DISRUPT CAD I study, S-IVL for heavily calcified coronary lesions was demonstrated to be a feasible first-line strategy in facilitating PCI via calcified plaque modification [3]. Delivery of all stents was achieved, with residual stenoses of less than 8.3%. The DISRUPT CAD II study provided further evidence on the safety of S-IVL in patients with severe CAC requiring coronary revascularization, with “high procedural success and minimal complications” [5]. There were no reported major dissections, perforations, closure, or flow complications. In-hospital and 30-day MACE were 5.8% and 7.6%, respectively [5]. The DISRUPT CAD II OCT sub-study analysis demonstrated S-IVL to significantly increase lumen area while decreasing calcium angle. S-IVL was not associated with the arterial injury or microembolization associated with atherectomy in the peri-procedural period. Importantly, when compared to atherectomy or specialty balloons, S-IVL is advantageous in its accessibility to interventionalists while lowering the risks of embolization associated with atherectomy. Additionally, S-IVL is not typically associated with guidewire bias or dependence on barometric pressures because of the circumferential plaque modification from the emitter. At low atmospheres, S-IVL also limits vascular injury. In fact, the DISRUPT CAD I and II trials have demonstrated the importance of S-IVL in reducing the extent of calcification in such lesions prior to PCI, in order to avoid stent malapposition and failure of expansion [3,5].

While S-IVL has been approved for CAD in Europe since May 2017, there is no current FDA approval in the US. The ongoing study DISRUPT CAD III is evaluating the safety and effectiveness of S-IVL for “de-novo, calcified stenotic coronary arteries prior to stenting,” as a staged pivotal study for the purpose of attaining FDA approval [6]. Wong et al. presented the use of S-IVL on the ostium of unprotected LMCA [7]. They also performed a small study of 26 patients; wherein S-IVL was used in 58% of patients upfront and 46% required further pre-dilatation with a NC balloon before stent deployment [7]. The same authors published a case series where S-IVL was used safely [8]. Venuti et al. reported varying plaque modification strategies in a patient who underwent multiple PCI and later underwent S-IVL-assisted PCI. Their patient had a positive outcome and was symptom-free at a three-month follow-up [9]. Further, Kassimis et al. discussed the fact that patients showing moderate to severe angiographic calcification and ≥ 270° calcium arc on intravascular imaging may benefit from S-IVL as an alternative to atherectomy [4].

In our case, conventional atherectomy presented a significant risk of poor outcomes, mostly because the involved site was located between two recently placed stents. Technical concerns included the potential disruption of stent struts from atherectomy devices and the possible dreaded retention or dislodgment of atherectomy burrs and crowns within these recently stented LMCA and LAD segments. Upon further review of this case, it appeared clear that the index management of the calcified complex plaque within the LMCA and LAD should have included the use of a priori atherectomy before stenting, in order to optimize plaque modification first. Yet, the patient was referred to our institution three months after the index procedure performed at a different facility, and we were then faced with the severe recurrent disease also involving under expanded stents in a high-risk coronary location and impacting a critically ill patient.

In established forms of intervention, perforation is a known complication with a documented rate of 0.9% in orbital atherectomy [4]. Excimer laser coronary atherectomy (ELCA) is an effective tool for the management of significant in-stent restenosis; however, its efficacy in severely calcified lesions is suboptimal [10]. Our patient’s extensive calcification was a significant barrier to adequate stent expansion. The use of S-IVL was therefore deemed appropriate in order to best optimize stent expansion, treat heavy intimal calcification (behind the existing stented struts), and improve luminal gain and distal flow. As described above, we used LV percutaneous support with the insertion of an impella CP probe and the successful use of S-IVL prior to conventional IVUS-assisted PCI. The combined use of these interventional and imaging modalities to perform this high-risk PCI resulted in a favorable clinical outcome. Clearly, the use of S-IVL in this case along with the growing body of literature regarding this technology suggests a new and accessible potentially first-line option for interventional cardiologists to facilitate successful coronary revascularization in patients with severely calcified coronary lesions and under expanded coronary stents. In conclusion, S-IVL represents a promising and effective new treatment modality for these disease conditions and adds to the existing interventional therapeutic armamentarium used to treat calcified lesions and under expanded stents.

## Conclusions

S-IVL is a novel method that may become a first-line option in the preparation of a lesion for stent deployment. The DISRUPT CAD trials have positioned S-IVL to change the current approach to heavily calcified CAD. The DISRUPT CAD III trial will be a driving force in adding S-IVL to the interventionalist’s toolkit with FDA approval. Data from further clinical trials will be required to further assess the safety, efficacy, and feasibility of S-IVL for CAD.
